# Review of Developments in Electronic, Clinical Data Collection, and Documentation Systems over the Last Decade – Are We Ready for Big Data in Routine Health Care?

**DOI:** 10.3389/fonc.2016.00075

**Published:** 2016-03-30

**Authors:** Kerstin A. Kessel, Stephanie E. Combs

**Affiliations:** ^1^Department of Radiation Oncology, Technische Universität München, Munich, Germany; ^2^Institute of Innovative Radiotherapy (iRT), Helmholtz Zentrum München, Neuherberg, Germany

**Keywords:** data collection system, electronic data capture, documentation system, data management system, Big Data

## Abstract

Recently, information availability has become more elaborate and widespread, and treatment decisions are based on a multitude of factors, including imaging, molecular or pathological markers, surgical results, and patient’s preference. In this context, the term “Big Data” evolved also in health care. The “hype” is heavily discussed in literature. In interdisciplinary medical specialties, such as radiation oncology, not only heterogeneous and voluminous amount of data must be evaluated but also spread in different styles across various information systems. Exactly this problem is also referred to in many ongoing discussions about Big Data – the “three V’s”: volume, velocity, and variety. We reviewed 895 articles extracted from the NCBI databases about current developments in electronic clinical data management systems and their further analysis or postprocessing procedures. Few articles show first ideas and ways to immediately make use of collected data, particularly imaging data. Many developments can be noticed in the field of clinical trial or analysis documentation, mobile devices for documentation, and genomics research. Using Big Data to advance medical research is definitely on the rise. Health care is perhaps the most comprehensive, important, and economically viable field of application.

## Introduction

In modern medicine, large data volumes, including imaging, treatment documentation, and follow-up information, are collected within the hospital or practice environment. Even in the age of intelligent information systems, doctors, nurses, and other health workers are faced with the difficulty of sharing data within the medical facility (1, 2). Thus, several groups have been working on various approaches solving this important task (3–5).

Recently, information availability has become more elaborate and widespread, and treatment decisions are based on a multitude of factors, including imaging, molecular or pathological markers, surgical results, and patient’s preference. In the past, paper-based documentation was the standard, which has been partially digitalized over the years, often leading to parallel worlds of documentation in one institution. As disease management steps into the era of modern personalized medicine (6), including various quantitative data, information becomes a strong focus, thus involving the active contribution of multiple medical specialties. Established structures to gather all significant data are therefore of high importance for reaching the best clinical performance and enhancing interdisciplinary and clinical research. Ultimately, this leads to the improvement, adaptation, and redevelopment of health-care concepts.

In interdisciplinary medical disciplines, not only heterogeneous and voluminous data must be evaluated but also spread across various information systems within several involved departments in a large variety of documentation styles (7, 8). Furthermore, in highly image intensive specialties, such as radiation oncology or radiology, diagnostic and therapeutic data acquisitions are acquired throughout the course of treatment and during follow-up. Clinicians and researchers need assistance in reusing the terabytes of invaluable information collected routinely into separate information systems (9). They hold hidden treasures (10). Exactly this concept is also referred to in many ongoing discussions about Big Data – the “three V’s”: volume, velocity, and variety (10). One could even add variability (inconsistency in data) and veracity (differences in data quality) as two more V’s equally important characteristics, especially in a medical context. To avoid double documentation, loss or mix-up of data, and to provide a fast and reliable basis to collect all relevant data, interconnected information systems have been developed (5).

The achievement of building systems merging all these specifications is a challenging task from both a technical and non-technical point of view. The focus must lie in providing flexibility and increasing performance for the future. This is associated with a vendor independent (6) and Integrating the Healthcare Enterprise (IHE) complying concept that strictly obeys given specifications for patient confidentiality and security mechanisms. Innovative methods and ideas are gaining ground in the field, which will be investigated by this analysis. We want to take a step back and perform a broad review of the developments in electronic clinical data management systems and the standards for data storage of the last decade, with special respect to the further processing of the collected data.

## Methods

Published data on the subject of clinical documentation and management systems within the last decade were searched for in all NCBI databases with specific inclusion/exclusion criteria. The terms for search were “((((data collection system[Title/Abstract]) OR electronic data capture[Title/Abstract]) OR documentation system[Title/Abstract]) OR data management system[Title/Abstract]) AND (‘2004/06/30’[Date – Publication]: ‘2014/06/30’[Date – Publication]).” We explicitly did not include the term “Big Data” to characterize the developments solely on clinical documentation of the last decade. The search delivered 895 hits. Subsequently, the following inclusion criteria were applied to the references: English or German language, topic of research, and medical specialty. Based on these criteria, 34 articles not written in English or German language were excluded from the analysis. We reviewed the articles and excluded further five articles, as they were not referring to any use or implementation of a data management system.

The review process was done by both authors. First, we reviewed the title and abstract of all articles. We looked at the topic of each paper and classified them in use or implementation of data management systems; comparison of new systems with a previous standard; or recommendations about system implementation and discussions about issues after system introduction.

Documentation and data management systems are used in many medical and biological specialties. It was not always possible to clearly determine the classification of an article. Particularly, interdisciplinary research activities across multiple disciplines and reveal a clear overlap between multiple topics. We obtained the main discipline of each paper and listed all those containing at least 15 articles. Furthermore, many articles contained insufficient information in the abstract some even had none. In this case, we read the whole paper to determine the topic of research and grouped each paper in a medical or biological specialty. In the final step, we carefully examined all papers containing descriptions about system implementation to find postprocessing ideas and concepts.

**Figure 1:**
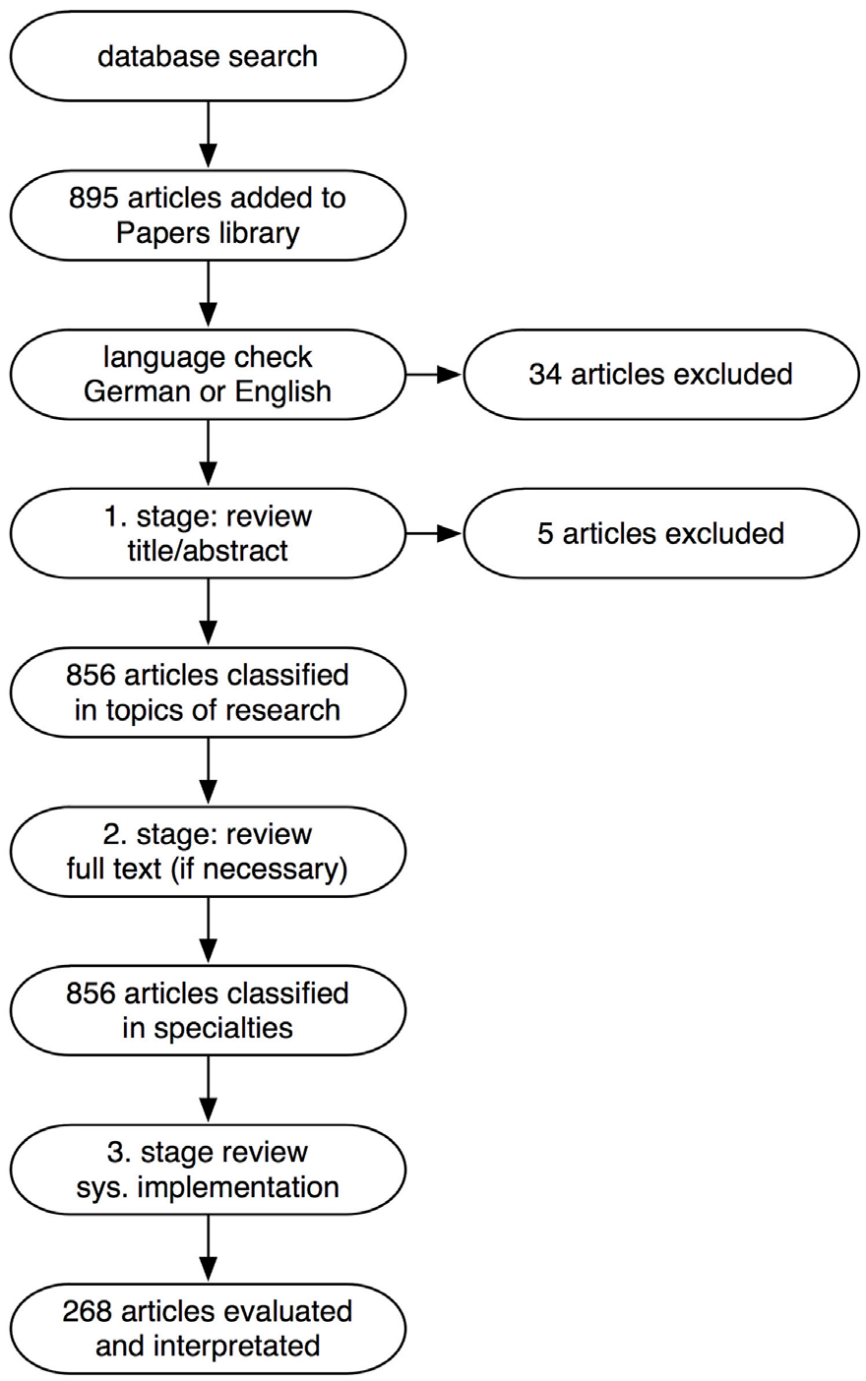
**Flow chart of the review methodology**.

A Papers 3 library (Mekentosj B.V., Amsterdam, Netherlands) was used to collect and organize the references. Figure 1 illustrates the overall review methodology.

## Results

Classification of articles into medical specialty and topic of research can be found in Tables 1 and 2, respectively.

**Table 1:** **Specialty of the articles**.

Specialty	No. of articles
Biology	96
Chronic disease management	67
Emergency and critical care medicine	63
Epidemiology	24
Health technology and medical informatics	95
Neuroscience	17
Nursing	109
Oncology	41
Palliative medicine	19
Pediatrics	22
Pharmacy	19
Psychiatry and psychotherapy	27
Public health	37
Surgery	31
Teaching	17
Other	172
Not assigned	39

	*N* = 895

**Table 2:** **Topics of articles**.

Topic	No. of articles
System use	469
• For clinical trial or analysis	370
• For clinical routine	99
System implementation	268
System comparisons with paper-based standard or other systems	24
System review, recommendations, and issues	95
Not assigned	39

	*N* = 895

### System Implementation

About one-third of all articles (*n* = 268) specifically discuss the development of a data collection system/database (as opposed to those referring to a system as a tool), and the results of implementing a data management system into the clinical environment.

Most of the developed systems provide data utilization through query, analytic, export, and reporting tools. These report and export functionalities are used with regard to statistical analyses, for example, by importing the data into a statistic software, such as SPSS, for further calculations. Only five of those articles discuss first ideas and ways to immediately make subsequent use of collected data, particularly imaging data (11, 12), for advanced analysis or postprocessing procedures beyond basic statistic analysis. Table 3 summarizes the work of these five research groups. The general criticism of the articles is that details about the implementations are vague and no general concept is presented, which could be transferred into another setting.

**Table 3:** **Articles with further processing strategies and approaches of collected data**.

Reference	Year	Summary
Brown et al. (16)	2007	Analysis tools connected to data management system for quantitative image analysis in metastatic lung cancer patients; automatic nodule detection and segmentation for CAD evaluation; communication standards used: DICOM
Carey et al. (17)	2012	Analysis tools used on imaging files stored in database in lung cancer patients; manual image analysis; no communication standardization mentioned
Haak et al. (11, 18)	2014	Analysis tools connected to EDC system for automatic image and biosignal analysis; communication standards used: web services, ODM, SOAP, SFTP, HTTP
Kessel et al. (12, 19)	2012	Analysis tools connected to documentation database *via* SQL interface; semiautomatic CT image registration and segmentation of pancreatic cancer patients, as well as dose calculation of radiation plans; communication standards used: HL7, DICOM, https
Ozyurt et al. (20)	2010	Analysis tools used on local copies of neuroimaging data after query and download from the data management system; results are transferred back *via* web services; communication standards used: web services, SOAP, DICOM, https

*CAD, computer-aided diagnosis; DICOM, digital imaging and communications in medicine; EDC, electronic data capture; HL7, health level 7; SQL, structured query language; ODM, object data model; SOAP, simple object access protocol*.

With the Big Data challenge, emerging data mining is a buzzword becoming more and more widespread, which is also reflected in recent articles in documentation and management systems (13–15).

### System Use

More than half of all articles (*n* = 469) mention the use of an electronic system, especially in clinical trial or analysis documentation (370/469). This trend is attributable to the many advantages, such as accessibility, backup, or central storage as opposed to paper-based documentation (5).

Many developments include mobile devices for documentation and making information available whenever wherever through web-based or app solutions. Especially in the field of nursing or chronic disease management, patients’ self-monitoring of health information takes place on web-based health platforms or apps (21–24). The use of mobile technologies in health care is also a trend in developing countries, where no global IT infrastructures but cellular networks are available (25–28).

### System Comparison and Review

Comparisons (24/469) and reviews (95/469) of systems are equally indicative: electronic data capture and documentation systems help in data gathering problems but lead to new problems on a technical and financial level.

**Figure 2:**
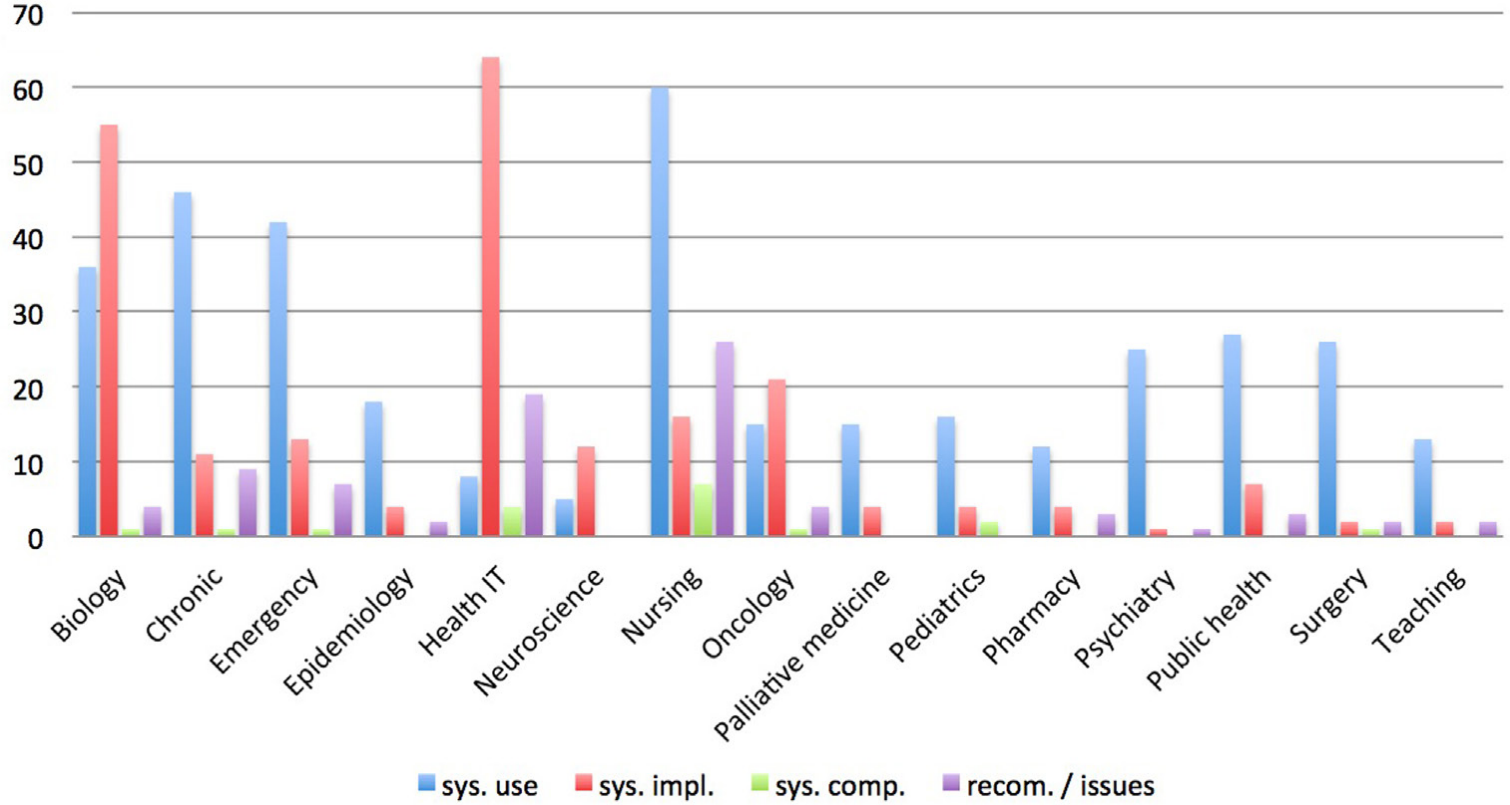
**Diagram showing the research topics distributed over the specialties**.

Figure 2 illustrates the research topics distributed over the specialties. Clearly, documentation supported by electronic systems in the areas of chronic disease management, nursing, and emergency medicine is successfully in use. These documentation systems maintain the daily recording of patient and treatment data, whereas in surgery, public health, epidemiology, and pharmacy documentation systems are mostly recording clinical trials and evaluations.

Naturally, the sector of health technology and medical informatics is most advanced in the development and implementation of documentation and management systems. Of equal relevance are the advances in biological genomics science. Here, numerous calculations are executed and massive amounts of data arise; hence, systemic data storage and management are essential. Many groups present their research environment, and an increasing interest in developing tools for further analysis can be noticed (13, 29, 30).

Recent advances and developments are currently made in the interdisciplinary disciplines, such as radiology, radiation oncology, and neuroscience (4, 12, 17, 20, 31–33). These involve various types of data, such as multimodal imaging, laboratory, and treatment data, which need to be correlated to analyze research questions and extract new information.

## Discussion

The Big Data challenge occupies all fields of science and economy. Just recently major companies, such as Google with Google Fit and Apple with HealthKit, started their platforms announcing “a health revolution” (34).

A lot of information increasingly accumulates. Automatic analyses are on the rise to manage this amount of data. Since literature on this topic is widespread and of varying quality, derives from several disciplines and misses detail to some extent, the aim of the present review is to summarize and classify reports on systems and implementation approaches to cope with the data challenge in medicine and to provide a basis for subsequent implementation strategies.

The tendency of having two (documentation) systems in a clinical facility is clearly visible (35, 36). On the one side is a clinical system, which can be an electronic health record (EHR) or hospital information system (HIS) in various designs used for routine and everyday patient and treatment documentation, on the other side, research systems are becoming established for scientific purposes (such as clinical trials, evaluations, and research data pool). Both data management systems go hand in hand, and structures are developed to share information, such as treatment and lab parameters, follow-up data and imaging, etc., between both and to avoid redundant data.

It is not the lack of technology or tools that keep “the health revolution” from coming, but the lack of expertise, specifications, and concepts (4). One of the most common weaknesses found is the lack of standardization. Most researchers create an individual in-house solution without considering communication standards, such as DICOM, HL7, https, and html (37). These solutions work only in their own environment and are tailored to meet their requirements. This might be necessary up to a certain level, as already stated that there is no “one-size-fits-all” solution for documentation of clinical trials, research data, or patient data *per se* (38). However, one must consider the further use of data, data sharing over time, and analysis procedures, which depend on standardized infrastructures and must comply with concepts of anonymization, liability, and data security. The importance lies in an interoperable approach – no “island solution.” Only coherent IT solutions bring sustainable and profound improvement of processes. It is up to us to enforce little known and little-established standards in health-care developments (3).

It may still be an idealized vision to be able to answer research questions in a medical department with a single mouse click. Many groups are working exactly with this aim in mind, but to date, only partial success can be reported. Based on the technology available, this seems to become possible in the future. The connection of analysis tools to a data management system and building an analysis pipeline is essential for this and the next logical step. However, an evaluation process depends highly on data resources. An effective data management is essential for any useful data analyses. Only with electronically captured, complete and high-quality data from the very beginning, conversion of data into new information delivers meaningful results.

In health-care environments, some scenarios have been demonstrated how automatic processing can be combined with manual interactions (5, 19). The concept to transfer this idea to an automatic workflow must consist mainly of two facts: (a) use current standards and work compliant to these standards and (b) build a central data pool that contains the “Big Data.” The idea can be summarized as ASER: acquire, store, exchange, and reuse of data.

Various techniques are currently underway, with simple object access protocol (SOAP) or web services only two mentioned, that provide functionality to attain that concept of combining analysis tools and execute them consecutively for an automatic analysis procedure (11, 39). Web services or services, in general, are characterized by their interoperability and their wide distribution even in the mobile world. They have the advantages to coordinate multiple tasks and at the same time be able to cope with large, heterogeneous data sets and high computation intensity. Handling heterogeneous, voluminous data sets is a fundamental requirement for working in an interdisciplinary environment, as already mentioned. Computer-aided diagnostics (CAD) applications could enable large amounts of data to be extracted for analyses as well.

Probably, more papers could be found with a database search focusing on analysis and postprocessing of data; however, the aim of the present work was to identify and review the current status of the connection of documentation and data management system in combination of subsequent analysis strategies.

The concept we propose is to take the next step and invest and build an intelligent infrastructure and craft complex algorithms. It should include a library of sophisticated analysis services/tools to be plugged together as needed for a specific research question, possibly in a way that it is usable for researchers with no or little IT knowledge to “make use of the Big Data” in health care. This way collaborative translational research will be effective and capable of handling all sorts of data. It already becomes its own profession to manage and coordinate Big Data having not only strong communication skills in an interdisciplinary environment but also multiple abilities such as knowledge about clinical processes, workflows, and underlying infrastructures as well as a strong scientific interest and IT background.

The visionary is already thinking about putting the Big Data into the cloud while most hospitals are still fighting with standard conform infrastructure. Still simple IT problems cause great difficulties in clinical routine, especially in large centers. However, the idea of Big Data analyses is tempting and would help us move personalized medicine forward. In summary, to answer our initial question, “Are we ready for Big Data in routine heath care?” we would answer no. Previously, we have reported on the details of our survey about data management in routine health-care environments (37). Only 7% stated that they are starting to develop solutions to cope with Big Data.

### Study Limitations

The research aim was to give a broad overview of the current status developments in electronic, clinical data collection, and documentation system. No specific aspects of data management systems are discussed.

## Conclusion

Using Big Data to advance medical research is now on the rise. Health care is perhaps the most comprehensive, important, and economically viable field of application. Adding meaning and context to Big Data can be achieved by investing in infrastructure and software and combining procedures to an analysis workflow. However, until now less experience is available on how to develop research questions that can be answered by such an infrastructure, and how to transfer the results into routine patient care. How soon are we able to incorporate it into decision-making?

## Author Contributions

KK performed the database search and review, drafted, and wrote the manuscript. SC revised the articles review as a second author. All authors read and approved the final manuscript.

## Conflict of Interest Statement

The authors declare that the research was conducted in the absence of any commercial or financial relationships that could be construed as a potential conflict of interest.
